# Review of Evidence for Adult Diabetic Ketoacidosis Management Protocols

**DOI:** 10.3389/fendo.2017.00106

**Published:** 2017-06-13

**Authors:** Tara T. T. Tran, Anthony Pease, Anna J. Wood, Jeffrey D. Zajac, Johan Mårtensson, Rinaldo Bellomo, Elif I. Ekinci

**Affiliations:** ^1^Department of Endocrinology, Austin Health, Melbourne, VIC, Australia; ^2^Department of Medicine, Austin Health, University of Melbourne, Melbourne, VIC, Australia; ^3^Department of Intensive Care, Austin Health, Melbourne, VIC, Australia; ^4^Menzies School of Health Research, Darwin, NT, Australia

**Keywords:** diabetic ketoacidosis, diabetes, insulin, rehydration, hypoglycemia, hypokalemia, metabolic acidosis, protocol

## Abstract

**Background:**

Diabetic ketoacidosis (DKA) is an endocrine emergency with associated risk of morbidity and mortality. Despite this, DKA management lacks strong evidence due to the absence of large randomised controlled trials (RCTs).

**Objective:**

To review existing studies investigating inpatient DKA management in adults, focusing on intravenous (IV) fluids; insulin administration; potassium, bicarbonate, and phosphate replacement; and DKA management protocols and impact of DKA resolution rates on outcomes.

**Methods:**

Ovid Medline searches were conducted with limits “all adult” and published between “1973 to current” applied. National consensus statements were also reviewed. Eligibility was determined by two reviewers’ assessment of title, abstract, and availability.

**Results:**

A total of 85 eligible articles published between 1973 and 2016 were reviewed. The salient findings were (i) Crystalloids are favoured over colloids though evidence is lacking. The preferred crystalloid and hydration rates remain contentious. (ii) IV infusion of regular human insulin is preferred over the subcutaneous route or rapid acting insulin analogues. Administering an initial IV insulin bolus before low-dose insulin infusions obviates the need for supplemental insulin. Consensus-statements recommend fixed weight-based over “sliding scale” insulin infusions although evidence is weak. (iii) Potassium replacement is imperative although no trials compare replacement rates. (iv) Bicarbonate replacement offers no benefit in DKA with pH > 6.9. In severe metabolic acidosis with pH < 6.9, there is lack of both data and consensus regarding bicarbonate administration. (v) There is no evidence that phosphate replacement offers outcome benefits. Guidelines consider replacement appropriate in patients with cardiac dysfunction, anaemia, respiratory depression, or phosphate levels <0.32 mmol/L. (vi) Upon resolution of DKA, subcutaneous insulin is recommended with IV insulin infusions ceased with an overlap of 1–2 h. (vii) DKA resolution rates are often used as end points in studies, despite a lack of evidence that rapid resolution improves outcome. (viii) Implementation of DKA protocols lacks strong evidence for adherence but may lead to improved clinical outcomes.

**Conclusion:**

There are major deficiencies in evidence for optimal management of DKA. Current practice is guided by weak evidence and consensus opinion. All aspects of DKA management require RCTs to affirm or redirect management and formulate consensus evidence-based practice to improve patient outcomes.

## Introduction

### Background

**Figure 1:**
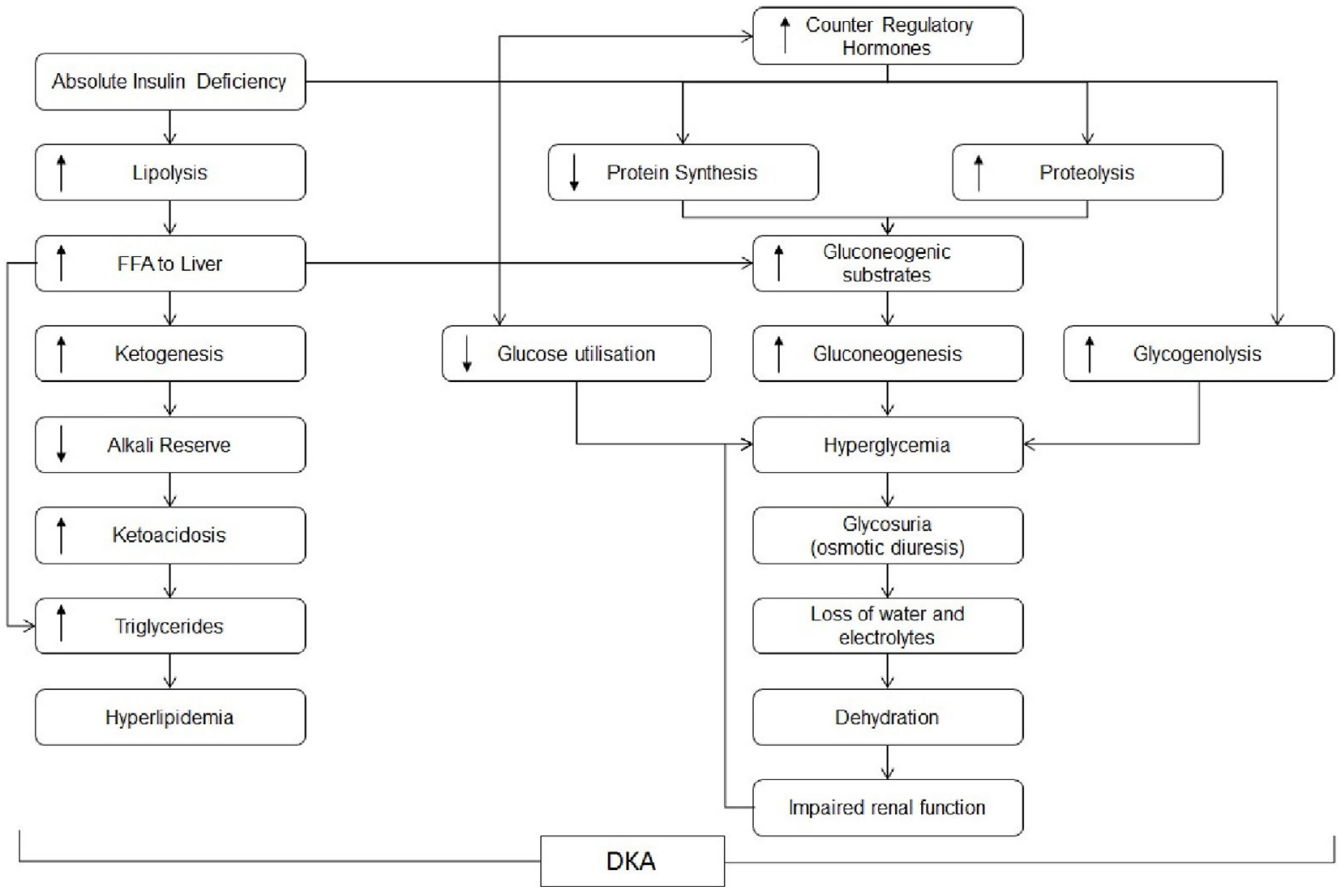
**Pathogenesis of diabetic ketoacidosis**.

Diabetic ketoacidosis (DKA) is characterised by the triad of hyperglycemia, ketosis, and metabolic acidosis. This results from a relative or absolute deficiency of insulin and an excess of counter-regulatory hormones including glucagon, cortisol, catecholamines, and growth hormones leading to hyperglycemia, glycosuria, dehydration, and hyperosmolarity of varying severity (Table 1) (1, 2). Glycosuria induces an osmotic diuresis, which results in significant deficits in fluid and electrolytes including sodium, potassium, calcium, magnesium, chloride, and phosphate (3). Dehydration and hyperglycemia results in hypertonicity and an efflux of water from the intracellular space to the hypertonic extracellular space. There is also a potassium efflux from the intracellular space, aggravated by acidosis, lack of effective insulin action, and breakdown of intracellular proteins (3) (Figure 1).

**Table 1:** **Definitions of severity of diabetic ketoacidosis (DKA)**.

	Mild DKA	Moderate DKA	Severe DKA
Arterial pH	7.25–7.3	7.0 to <7.24	<7.0
Serum bicarbonate (mmol/L)	15–18	10 to <15	<10
Anion gap	>10	>12	>10
Mental status	Alert	Alert/drowsy	Stupor/coma

*Adapted from American Diabetes Association 2009 consensus statement. In all cases, plasma glucose is >14 mmol/L (>250 mg/dL), urine, and serum ketones are positive and effective osmolality is variable*.

Diabetic ketoacidosis more often complicates type 1 rather than type 2 diabetes mellitus and carries the risk of significant morbidity and mortality (1, 4, 5). Despite evolving practice, there are increasing numbers of hospital admissions for DKA and hyperosmolar hyperglycaemic state (HHS) (1, 6, 7). DKA is associated with mortality rates as high as 5–9% in the elderly and in patients with severe comorbidities (1, 8). Though mortality from DKA is more often attributable to severe underlying illness and comorbidities (1), DKA itself is a hypercoagulable state resulting in potentially fatal complications including stroke, myocardial infarction, and disseminated intravascular coagulation (9, 10). Management involves rehydration, correction of electrolyte derangements; particularly hypokalaemia, administration of insulin, correction of metabolic acidosis, and treatment of precipitants such as infection, pancreatitis, trauma, and myocardial infarction (11–13). Complications of DKA management include pulmonary venous congestion and severe electrolyte imbalance. Cerebral oedema represents a major potential complication, although this has largely been demonstrated in children.

### Aims of Review

This review is intended to assist those writing and utilising DKA management protocols in adults to appreciate deficits in current knowledge and to draw attention to areas that may benefit from future research.

We reviewed the original studies considering key elements of inpatient management of DKA including the choice of intravenous fluids and rates of replacement; insulin infusion rates, and routes of administration; potassium replacement rates; and the role of bicarbonate and phosphate replacement.

As many institutions use protocol driven management, we considered the role of protocols by evaluating available audits of such documents to determine whether these protocols significantly alter clinician decisions, patient outcomes, and hospitalisation duration. We also addressed the correlation of DKA correction rate on patient outcomes.

## Methods

**Figure 2:**
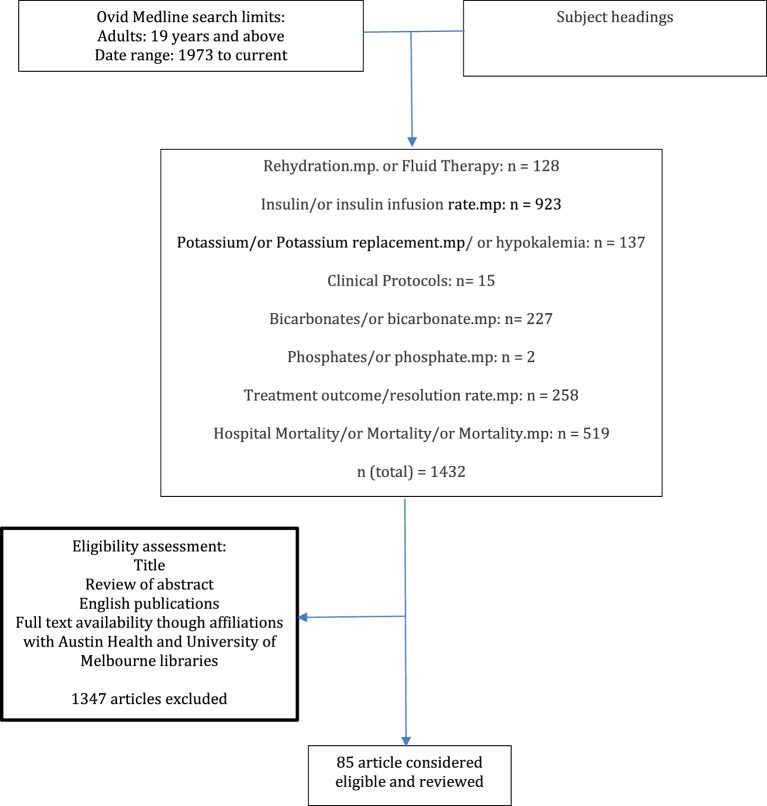
**Study selection flow diagram**.

From January 3, 2015, until April 11, 2016, two review authors: AP and TT performed Ovid Medline searches combining *Subject Headings* (Diabetic Ketoacidosis.mp. or Diabetic Ketoacidosis/), with key words of different DKA management aspects, with the applied limits: “all adult” (19 plus years), published from “1973 to current” (Figure 2).

## Results

Ovid Medline searches yielded 1,432 articles in addition to those accessible from reference lists of national consensus statements. Eligibility was determined by two reviewers’ assessment of title, abstract, and full-text/print availability through affiliations with Austin Health’s and the University of Melbourne’s libraries. 85 articles published between 1973 and 2016 were considered eligible and were reviewed (Figure 2).

### Hydration

Patients with DKA experience osmotic diuresis, resulting in hyperosmolar intracellular dehydration (14). Fluid deficits may be up to 10% of total body weight (1, 5). There is also an accumulation of β-hydroxybutyrate and acetoacetate, which results in a high anion gap metabolic acidosis (1). Prompt rehydration is vital to restore circulating volume and tissue perfusion, clear ketones, and correct electrolyte imbalances (1, 5, 14). Independent of insulin therapy, hydration alone restores circulatory volume and tissue perfusion; improves glycaemic control and acid base balance, and reduces counterregulatory hormones (5, 14).

#### Hydration—Choice of Fluids

Isotonic fluids have been established as the preferred fluid choice since a pivotal study in 1958 compared with hypotonic, isotonic, and hypertonic fluids in patients with severe DKA. These authors found that hypertonic fluids may be detrimental due to worsening of hyperosmolarity, hypernatraemia, and hyperchloraemia, while hypotonic fluids were noted to cause diuresis (3).

In 2013, a Cochrane review of 78 randomised controlled trials (RCTs) assessed the effect of colloids compared to crystalloids for fluid resuscitation in critically unwell patients with trauma, burns or in the post-operative period (15). Crystalloids were preferred as colloids were considerably more expensive and offered no survival benefit over crystalloids (15). However, specifically hydroxyethyl starch crystalloids were associated with a significant increase in mortality and acute kidney injury, and it was concluded that they should be avoided (14–19). It is important to note that none of the included studies specifically considered these fluids in the setting of DKA.

The optimal crystalloid solution to use in DKA is unclear (Table 2). Among crystalloids, normal saline (0.9% sodium chloride) has come under scrutiny due to concerns raised over hyperchloraemic metabolic acidosis, as well as a possible propensity for oliguria, prolonged acidosis, and coagulopathy (1, 5, 14, 16–18, 20–25).

**Table 2:** **Choice of crystalloids for rehydration in adult patients with diabetic ketoacidosis**.

Study	Control arm	Study arm	Results	Conclusion
Mahler et al.—randomised double-blind prospective trial (16)	Normal saline (*n* = 23)	Plasma-Lyte (*n* = 22)	Mean increase in chloride: 16.5 mmol/L (95% CI = 14–19) normal saline group 8 mmol/L (95% CI 6–9) in Plasma-Lyte group (*p* ≤ 0.001)	Use of BES prevents hyperchloraemic metabolic acidosis
			Mean increase in bicarbonate: 7 mmol/L (95% CI 5–8) normal saline group 9 mmol/L (95% CI = 8–11) Plasma-Lyte group (*p* = 0.023)	

Chua et al.—multicenter retrospective study (14)	Normal saline (*n* = 14)	Plasma-Lyte (*n* = 9)	Median serum bicarbonate correction higher in the Plasma-Lyte group at: 4–6 h (8.4 vs. 1.7 mEq/L) and at 6–12 h (12.8 vs. 6.2 mEq/L) from baseline (*p* < 0.05)	BES offers earlier metabolic correction No difference in clinical outcome
			Median standard base excess improved by: 10.5 vs. 4.2 mEq/L at 4–6 h 16.0 vs. 9.1 mEq/L at 6–12 h in the Plasma-Lyte and normal saline group, respectively (*p* < 0.05)	

Van Zyl et al.—RCT (26)	Normal saline (*n* = 29)	Ringers lactate (*n* = 28)	Median time to reach pH 7.32 Normal saline group: 683 min (95% CI 378–988) Ringers lactate group: 540 min (95% CI 184–896) (*p* = 0.251)	No evidence for benefit in use of ringers lactate
			Time to blood glucose: 14 mmol/L Normal saline: 300 min (IQR 235–420) Ringers lactate: 410 min (IQR 240–540) (*p* = 0.044)	

Bellomo et al.—prospective open-label, sequential period pilot study 2012 (27)	Chloride liberal (*n* = 760)	Chloride restricted (*n* = 773)	Mean serum creatinine increase: Chloride liberal: 22.6 µmol/L (95% CI 17.5–27.7 µmol/L) Chloride liberal 14.8 µmol/L (95% CI 9.8–19.9 µmol/L) (*p* = 0.03)	Chloride restrictive strategy decreases incidence of AKI and use of RRT No difference in hospital mortality, hospital or ICU length of stay, or need for RRT after hospital discharge
			AKI (RIFLE defined) Chloride liberal 14% (95% CI 11–16%; *n* = 105) Chloride restricted 8.4% (95% CI 6.4–10%; *n* = 65) (*p* = < 0.001)	
			RRT: OR 0.52 (95% CI 0.33–0.81) (*p* = 0.004)	

*Chloride liberal: normal saline, 4% succinylated gelatine solution, and 4% albumin*.

*Chloride restricted: Hartmann’s solution, balanced electrolyte solution (Plasma-Lyte 148), 20% albumin*.

*RRT, renal replacement therapy; RCT, randomised controlled trial*.

During the recovery phase of DKA, hyperchloraemia tends to develop because of preferential excretion of ketones during rehydration and improved renal perfusion, resulting in a raised anion gap metabolic acidosis (14). It is proposed that rehydration with normal saline may contribute to hyperchloraemia and a hyperchloraemic metabolic acidosis with a persisting base deficit and may cause renal vasoconstriction and decreased glomerular filtration rate (14, 27). Alternatively, this acidosis may represent a physiological response to resolving DKA rather than a result of the hydration fluid itself (17, 20–23, 28).

In the DKA-specific setting, a small, randomised, double–blinded, prospective trial (*n* = 45) compared the effects of fluid resuscitation in patients with DKA using Plasma-Lyte (a balanced electrolyte solution) and normal saline and concluded that balanced electrolyte solutions resulted in lower serum chloride and higher bicarbonate levels consistent with prevention of hyperchloraemic metabolic acidosis (16). However, there was no difference in clinical outcomes (16) (Table 2).

A similar conclusion was drawn in a small three centre retrospective study (*n* = 23) which compared Plasma-Lyte to normal saline as fluid resuscitation in DKA (14). This study was limited by its patient numbers with difficulty matching cases to controls.

The only other RCT was also small, studied different crystalloids in DKA, comparing patients who received Ringer’s lactate (a balanced electrolyte solution) (*n* = 28) with patients who received normal saline (*n* = 29). In this study, Ringer’s lactate offered no significant superiority in time to normalisation of pH and took a significantly longer time to achieve a blood glucose concentration ≤14mmol/L (26). A proposed mechanism for this delay in control of blood glucose level was that lactate from Ringer’s solution provided excess substrate for ongoing gluconeogenesis (26). Using the 2006 (and then the 2009 *post hoc*) American Diabetes Association (ADA) definitions for resolution of DKA, fluid selection made no statistically significant difference in time to resolution (26).

Another concern regarding normal saline is saline-induced oliguria. In the DKA context, a RCT comparing Plasma-Lyte to normal saline found cumulative urine output to be lower at 4–6 h in the normal saline group (14).

Balanced electrolyte solutions have insufficient potassium content when used alone and are not available with adequate premixed potassium (7). For this reason, the United Kingdom National Institute for Health Care Excellence (NICE) and the ADA guidelines favour continued use of normal saline (29).

From the available evidence, we conclude that there is no evidence for significant superiority between crystalloid and colloid solutions, while noting that hydroxyethyl starch solutions are best avoided due to risk of mortality and acute kidney injury.

**Figure 3:**
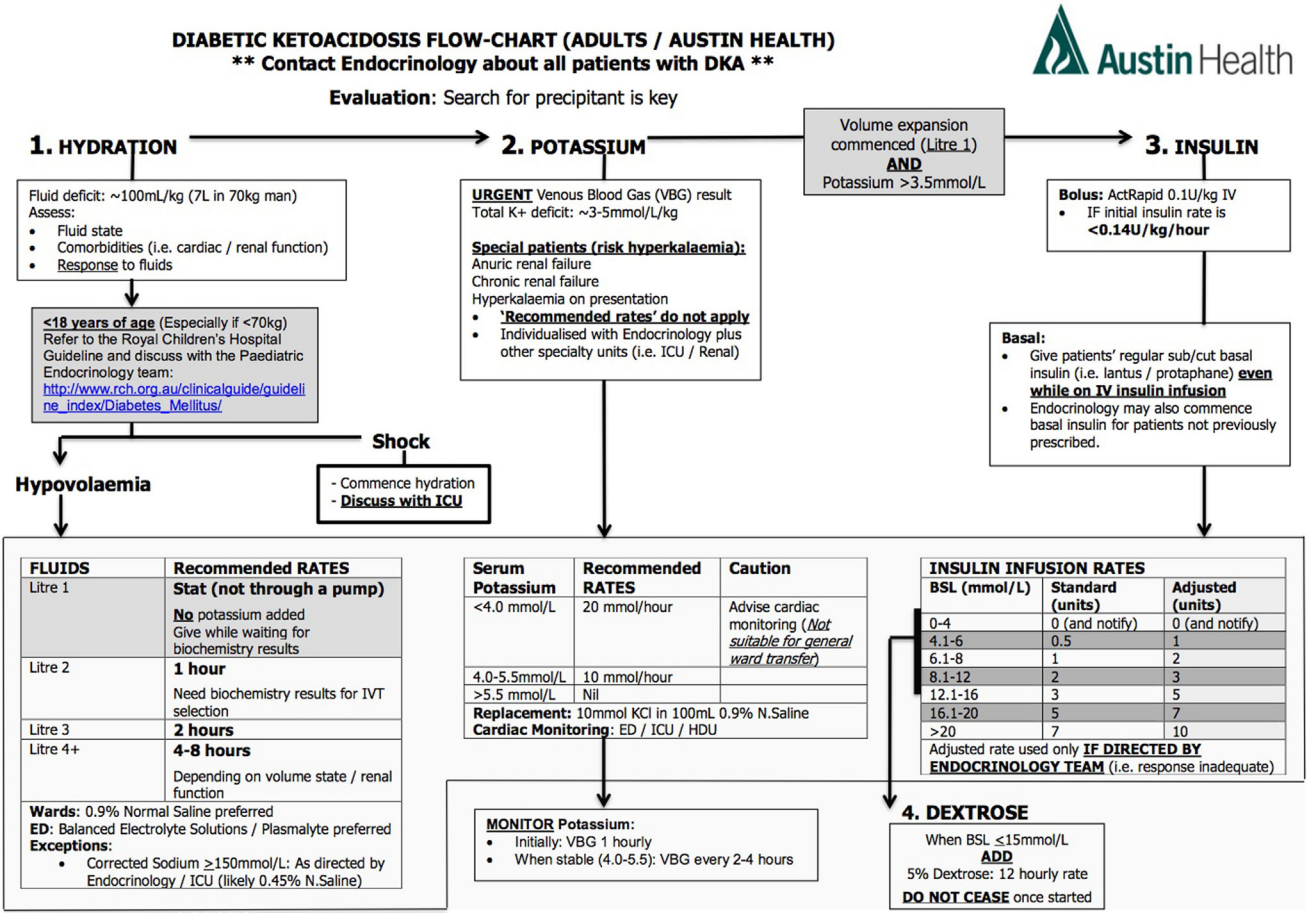
**Protocol for management of diabetic ketoacidosis at our centre**.

There is currently insufficient evidence for superiority amongst crystalloids, with very weak evidence to suggest that balanced electrolyte solutions may reduce the risk of hyperchloremic metabolic acidosis, though this has not translated to any clinical significance in patient outcomes in DKA. Normal saline is cheap and familiar to all clinicians and therefore can be used as the fluid of choice (Figure 3).

#### Hydration—Rate of Replacement

The rationale for intensive hydration in DKA is to avoid hypoperfusion, correct marked hyperglycaemia and hyperosmolarity, and improve responses to insulin therapy (1). Alternatively, the rationale for slower rehydration is to reduce the risks associated with correcting the hyperosmolar state too rapidly (30, 31) thus decreasing the potential risk of cerebral oedema (31, 32) as reported in children (32, 33).

Most studies of hydration rate take place in the intensive care setting making use of central venous pressure monitoring as opposed to general ward-based management. The latter is more common in the Australian context (22, 28, 34, 35).

Conservative hydration in relatively small numbers of open label studies revealed no adverse events, although the selection of patients was tightly regulated and may not be generalisable (22, 23, 28, 35). In 1989, a prospective analysis (*n* = 28) assessed aggressive resuscitation at 1,000 mL/h for 4 h followed by 500 mL/h for 4 h, compared to rehydration at half this rate (22). The study concluded that a slower rate of rehydration still led to prompt recovery, lack of any harmful effects, and had a significant reduction in the overall cost of medical therapy (22). This principle was reiterated by another, also prospective randomised controlled study with limited numbers (*n* = 27), comparing the effect of hydration using a rate of 1,000 mL/h versus a rate of 500 mL/h of normal saline, which concluded there was no significant difference in rate of resolution of biochemical derangements (36).

The major concern with rapid hydration is cerebral oedema which is seen in the paediatric population (33) and rarely reported in adult literature. There have been limited numbers of prospective trials, although given the rarity of this outcome, these have been inadequately powered (16, 20–23, 28, 35). Others have argued that rapid hydration is not a concern as cerebral oedema, as demonstrated by CT imaging of the brain, may be present prior to hydration and relates to the severity of initial acidosis rather than medical intervention (31, 34).

Pulmonary venous congestion is another potential complication of DKA management, although there is minimal evidence for this occurrence. Assessing extravascular lung water and colloid-osmotic pressure in a case series from 1988 did not reveal any tendency towards pulmonary venous congestion, despite large total infused volumes of 6 ± 1.01 L (35).

The ADA consensus statement in 2009 recommended that in the absence of cardiac compromise, normal saline is to be infused at a rate of 15–20 mL/kg body weight or at 1–1.5 L during the first hour (1). Determination of subsequent rates should be dependent on patient haemodynamic and hydration status and serum electrolyte levels (1). Neither studies nor guidelines addressed the issue of fluid replacement in obese patients with DKA.

In patients with normal or high corrected serum sodium levels, the recommended rehydration fluid is 1/2 normal saline (0.45% sodium chloride) given at 250–500 mL/h, whilst the recommended choice of rehydration fluid for patients with low corrected serum sodium is still normal saline, infused at the same rate (1). In either instance, fluid deficits should be corrected within the initial 24-h period (1). NICE guidelines recommend hydration at rates which are “not too rapid” except in cases of circulatory collapse (29).

The currently available evidence is very weak and suggests slow hydration rates result in equal management outcomes, with no consensus regarding optimal rates.

### Potassium Replacement—Dose and Rates

Patients with DKA are often found to initially have mild to moderate hyperkalaemia, despite a total body deficit of potassium (1). The initiation of insulin causes an intracellular shift of potassium and lowers the potassium concentration potentially resulting in severe hypokalaemia. Hence patients with serum potassium levels <3.3mmol/L need initial management with fluid resuscitation and potassium replacement, whilst delaying commencement of insulin until after potassium levels are above 3.3 mmol/L, to avoid cardiac arrhythmias, arrest, and respiratory muscle weakness (2).

Published trials use a range of potassium replacement rates from 10 to 40 mmol/h, which have not been reported to lead to adverse events. However, it is noted that potassium infusion rates and potential complications from over-replacement of potassium were not always analysed as outcomes (1, 5, 24, 37–40). These studies were not designed to determine optimal rates of potassium replacement or the required care settings and cardiac monitoring associated with different rates of potassium replacement during treatment of DKA, although it has been recommended that hypokalaemia (below 3.5 mmol/L) warrants admission to a high dependency unit (HDU) or equivalent (5).

Of note, the ADA guidelines suggest the addition of 20–30 mmol of potassium in each litre of infusion fluid to maintain target “normokalaemia” defined as a potassium of 4–5 mmol/L (1, 5). This is in contrast to the Joint British Diabetes Societies’ guideline recommending a replacement dose of potassium of 40 mmol, premixed in infusion fluid, if serum potassium is less than 5.5 mmol/L and the patient is passing urine, whilst faster replacement is to be guided by local protocols (5).

Currently, there is lack of evidence guiding the ideal potassium replacement rate, the associated necessary cardiac monitoring and care settings with guidelines recommending rates between 20 and 40 mmol in 1 L of rehydration fluid.

### Bicarbonate

Significant controversy surrounds the role of bicarbonate replacement in DKA. Some argue that correcting ketosis with insulin is adequate for reversing acidosis (1, 2, 7, 41). Others argue that bicarbonate therapy is warranted given the complications of severe metabolic acidosis (1, 2) (Table 3).

**Table 3:** **Complications associated with biochemical derangement and their correction**.

Severe hypokalemia	Potassium over-replacement
Severe muscle weakness or rhabdomyolysis	Muscle weakness and paralysis
Cardiac conduction abnormalities	Cardiac conduction abnormalities
Fatal arrhythmias (VT, VF)	Fatal arrhythmias (VT, VF, sinus arrest, asystole)

**Severe metabolic acidosis**	**Bicarbonate replacement**

Impaired myocardial contractility	Hypokalaemia
Cerebral vasodilation	Decreased tissue oxygen uptake (1)
Gastrointestinal complications	Cerebral oedema in children (1, 14)
Coma (1, 2)	Paradoxical worsening of ketosis in adults (14)

**Severe phosphate deficiency**	**Phosphate over-replacement**

Respiratory and skeletal muscle weakness, Haemolytic anaemia and poor cardiac contractility (2, 41)	Hypocalcaemia (1)

*VT, ventricular tachycardia; VF, ventricular fibrillation*.

Small randomised controlled studies demonstrate that bicarbonate can improve acidosis resolution transiently in DKA with pH 6.9–7.1 (14, 45) although this did not translate to improvement in morbidity, mortality, or time to recovery from hyperglycaemia, ketoacidosis, cardiac, or neurological outcomes (1, 2, 45). Conversely, bicarbonate replacement has its own associated complications (1, 14) (Table 3). Furthermore, there have been no prospective randomised studies investigating the impact of administering bicarbonate in patients with pH < 6.9 (2).

Due to the known complications of severe acidosis (Table 3), the ADA consensus guidelines that recommend patients with pH < 6.9 should be administered 100 mmol of sodium bicarbonate with 20 mmol potassium chloride/h for 2 h until venous pH is >7.0 (1, 2).

It appears clear that bicarbonate replacement offers no benefit when pH > 6.9. For cases of pH < 6.9, whilst there is lack of evidence, there is recommendation based only on expert opinion that replacement should be considered.

### Phosphate

In DKA, serum phosphate often appears normal or even increased, although there is a total body deficit of phosphate, typically up to 1.0 mmol/kg body weight which further decreases with insulin therapy (1, 2). Complications of phosphate deficiency are uncommon but may occur in severe cases (2, 41) (Table 3).

Prospective randomised studies demonstrated that phosphate replacement offers no improvement to DKA outcomes (1). Acknowledging this, the ADA consensus statement suggests that phosphate replacement with 20–30 mmol of potassium phosphate added to replacement fluid may be appropriate in patients with cardiac dysfunction, anaemia, respiratory depression, or patients with phosphate levels <3.2mmol/L (1). This contrasts against NICE guidelines that recommend against phosphate replacement (29).

### Insulin Administration

Once hypokalaemia is corrected and hydration commenced, insulin should be administered to halt lipolysis, ketogenesis, and correct hyperglycaemia. Regular insulin is favoured over insulin analogues. The current mainstay of insulin therapy in DKA is continuous intravenous infusion for its rapid onset and ease of dose titration (1, 2). Some institutions require intravenous insulin infusions to be managed in the intensive care setting and thus some advocate for the use of subcutaneous or intramuscular injections in order to avoid an intensive care admission (1, 46–48).

#### Insulin—Choice of Insulin

In 1987, Storms et al established that regular human insulin was superior to porcine insulin due to faster resolution of DKA (49), and for decades regular human insulin has remained the insulin of choice in DKA management. This convention has been challenged by the emergence of short and ultrashort-acting insulin analogues, which has now become much more familiar to clinicians than regular human insulin in general diabetes management.

Insulin analogs glulisine, aspart and lispro have been reported to have equal efficacy and *in vivo* potency compared to regular insulin in animals and humans, attributable to their similar receptor binding affinity and receptor mediated clearance (50–53), although only studies comparing the role of intravenous glulisine infusion as an alternative to intravenous infusion of regular human insulin has been performed (54). In spite of their more rapid onset, studies comparing the pharmacokinetics and pharmacodynamics of intravenous glulisine (an ultrashort-acting insulin analogue) to intravenous regular insulin (short-acting insulin) have found glulisine demonstrates equivalent glucose utilisation and disposal; and a similar distribution and elimination profile to regular insulin (50, 54).

Non-inferiority was demonstrated in a small, multicentre, open-label RCT (*n* = 68), comparing IV infusions of glulisine to regular human insulin. This study found both insulin types to be equally effective during the acute treatment of DKA, with no statistical difference in mean treatment duration (15.7 ± 4.5 vs. 20.5 ± 12 h) or amount of insulin infused until resolution of DKA (70 ± 33 vs. 76 ± 46 units) for glulisine and regular human insulin, respectively (54). Furthermore, the rate of decline of blood glucose concentrations, changes in bicarbonate and anion gap were similar, and neither groups experienced mortality or recurrence of DKA.

Due to the lack of superiority and the higher cost of insulin analogs, IV infusion of regular insulin is still considered the mainstay over insulin analogs in ADA consensus statement, though has not been specified in the NICE guidelines (1).

#### Insulin—Route of Administration

The attraction of subcutaneous insulin is to avoid the need for intensive care unit admission for IV insulin infusions and its associated high costs, although the concern is that it may not be as effective as IV insulin.

Studies comparing treatment of DKA using subcutaneous or intramuscular insulin against conventional IV insulin infusions found slower plasma glucose reductions at 1 h (failure to reduce by 10%) (55) and at 3 h (56) and slower ketone clearance (56).

Additionally, a retrospective study (*n* = 59) comparing bolus insulin injections in intravenous, subcutaneous, and intramuscular routes against continuous IV infusions found a higher rate of hypoglycemic episodes amongst the bolus insulin injection group (8 out of 30 patients), compared to the intravenous infusion group (1 out of 29 patients) (*p* = 0.03) (57).

Importantly, none of the abovementioned studies assessed longer term outcomes such as time in intensive care or length of hospital stay. As such, it is unclear whether rapid correction of hyperglycemia and ketonaemia necessarily leads to better outcomes.

A Cochrane review in 2016 analysed five small RCTs (total *n* = 201) comparing subcutaneous injections of insulin analogues (lispro and aspart) to regular insulin IV infusions in patients with mild to moderate DKA (Table 4) and revealed no statistical difference in time to resolution of DKA or rates of hypoglycaemia (42). These findings were based on very low to low-quality evidence and were limited by the number of included trials and participants. Other than hypoglycaemia, there was no analysis of adverse outcomes, and no deaths occurred in these studies (42).

**Table 4:** **Impact of insulin administration routes on diabetic ketoacidosis**.

Insulin types	Outcomes measured	Effect size	Number of participants and trials
SC insulin lispro vs. IV regular insulin	Mean difference in time to resolution of DKA	0.2 h (95% CI −1.7 to 2.1) (*p* = 0.81)	(*n* = 90, 2 trials)
SC insulin aspart vs. IV regular insulin	Mean difference in time to resolution of DKA	−1 h (95% CI −3.2 to 1.2) (*p* = 0.36)	(*n* = 30, 1 trial)
SC insulin lispro vs. IV regular insulin	Hypoglycemic events	Ratio of 0.59 (95% CI 0.23–1.52) (*p* = 0.28)	(*n* = 156, 4 trials)
SC insulin aspart vs. IV regular insulin	Risk of hypoglycaemic episodes	Risk ratio 1.00 (95% CI 0.07–14.55) (*p* = 1.0)	(*n* = 30, single trial)
Insulin lispro vs. IV regular insulin	Difference in mean hospital length of stay	−0.4 days (95% CI −1 to 0.2) (*p* = 0.22)	(*n* = 90, 2 trials)
SC insulin aspart vs. IV regular insulin	Difference in mean length of stay	1.1 days (95% CI −3.3 to 1.1) (*p* = 0.32)	(*n* = 30, single trial)

*Extracted from Cochrane Database of Systematic Reviews 2016 (42)*.

The benefits of administering subcutaneous insulin injections hourly and 2 hourly outside of the intensive care setting must also be weighed against the increased demands on nursing staff on medical wards, increased variability with dose administration times, time to onset of action, peak effect, and duration of effect (58).

An alternative management strategy is the initiation of both an intravenous insulin and continuation of the patient’s subcutaneous long-acting insulin during the initial management of DKA. This aims to decrease insulin infusion requirements, provide background insulin once intravenous insulin is discontinued, and reduce the risk of hypoglycaemia postcessation of insulin infusion. This was recommended in the NICE guidelines 2016 but was not present in the ADA 2009 consensus statement (1). This concept is supported by a prospective randomised study, which concluded that once daily subcutaneous insulin glargine administered during intravenous insulin infusions, safely prevented rebound hyperglycaemia without risk of hypoglycaemia (43).

Conversely, a pilot prospective RCT concluded that this model of treatment was feasible, although it did not find a significant benefit in time to closure of anion gap, or number of hypoglycaemic events (44) (Table 5).

**Table 5:** **Outcomes of combining subcutaneous insulin with insulin infusion**.

Trial	Arms	Outcomes of IV insulin infusion vs. IV insulin infusion plus glargine retrospectively	*p*-Value
Hsia et al.—prospective randomised study (43)	IV Insulin infusion (*n* = 31) vs. IV insulin infusion plus glargine^a^ (*n* = 30)	BGL > 10 mmol/L at 12 h follow-up: 29 (93.5%) vs. 10 (33.3%)	<0.001
		Hypoglycemic events: 9.6 vs. 0.0%	Not stated

Doshi et al.—pilot prospective RCT (44)	IV insulin infusion (*n* = 20) vs. IV insulin infusion plus glargine (*n* = 20)	Time to closure of anion gap (TCAG): 11.6 (±6.4) vs. 10.2 h (±6.8)	0.63
		Length of hospital stay: 4.6 (± 3.6) vs. 3.9 days (± 3.4)	0.66
		Number of hypoglycaemic events: 3 (15%) vs. 2 (10%)	1.0

*^a^Glargine dosed at 0.25 U/kg per body weight*.

*RCT, randomised controlled trial*.

Since the first trials in the 1970s, continuous subcutaneous insulin infusions delivered via insulin pumps have been used in patients with type 1 diabetes, and it is well established that DKA can occur as a complication of pump failure (59–62). Currently, there is no evidence surrounding the continuation or discontinuation of pump therapy during management of DKA. Current practice involves cessation of insulin pump therapy during management of DKA, then transition back to pump therapy after resolution of DKA (60). Conversely, given the potential roles of both continuous intravenous insulin and subcutaneous insulin injections, it could be postulated that insulin pumps may have a role in DKA management, as it is a convenient and pre-existing route of administration, although no studies have explored this potential.

The ADA 2009 consensus statement asserts that insulin therapy is effective regardless of route of administration, although both the ADA and NICE guidelines advocate for intravenous infusion (1, 29). The current evidence is weakly suggestive that there is no difference in outcome between administration of insulin as IV infusion or SC injections and is conflicting regarding impact of combining SC long-acting insulin with IV insulin infusion. Morbidity and safety data are limited with hypoglycemia being the only reported adverse outcome, and no deaths occurred in studies to date.

#### Insulin—Role of Initial Intravenous Insulin Bolus

An initial intravenous insulin bolus has been considered in multiple small trials.

A small prospective analysis in 1995 found patients who received an insulin bolus had a significantly higher incidence of hypoglycemia but no difference in hypokalaemia (57).

In contrast, a prospective observational cohort study in 2007 (*n* = 157) found no statistical difference in the incidence of hypoglycaemia, rate of glucose reduction, or length of hospital stay (63) and concluded that an initial bolus of intravenous insulin offered no significant clinical benefit or harm. The study was limited by lack of randomisation; standardised treatment protocols and used administration of 50% dextrose as a surrogate definition for hypoglycemia rather than serum glucose levels.

In 2008, a small prospective randomised protocol established that an initial bolus of insulin avoided the need for supplemental insulin doses if the insulin infusion rate was less than 0.14 units/kg/h (1, 64). The study compared patients (*n* = 12) who received a loading dose of 0.07 units/kg of regular insulin, followed by 0.07 units/kg/h insulin infusion, group 2 (*n* = 12) insulin infusion at 0.07 units/kg/h without loading insulin and group 3 (*n* = 13) insulin infusion at 0.14 units/kg/h, without loading. It found that group 2 patients required supplemental insulin doses, whilst other patients did not. Otherwise, there was no significant difference in duration taken to reach a serum resolution of DKA, length of hospital stay, hypokalaemia, or other complications including death (64).

Despite these findings, use of an initial bolus insulin dose has been incorporated into the ADA 2009 consensus statements but not in the NICE guidelines (29). The limited available evidence suggests use of bolus insulin results in no difference in outcomes.

#### Insulin Dose

Three decades ago, a small prospective RCT (*n* = 48) established the preference for low-dose insulin over high-dose insulin in management of hyperglycaemic emergencies (65). The study concluded there was no difference in correction rate of biochemical derangements, though 25–50% of patients who received high-dose insulin developed hypoglycaemia or hypokalaemia, compared to only 0–4% rate amongst patients who received low-dose insulin (3, 5, 65).

The intravenous insulin rates remain contentious, with fixed weight-based insulin rates being advocated by national authorities (1, 5, 24), to account for changing patient demographic, including obesity and higher insulin-resistance states such as pregnancy (5).

The Joint British Diabetes Society guidelines recommend insulin infusion at a rate that achieves a reduction in blood ketones by at least 0.5 mmol/L/h or alternatively, a venous bicarbonate increase by 3 mmol/L/h and capillary blood glucose reduction by 3 mmol/L/h (5). Similarly, the 2009 ADA consensus statement recommend an insulin infusion rate adjusted to target a serum glucose reduction rate of 3–4 mmol/L/h with no suggestions for target ketone reduction (1), once serum glucose levels reach 11.1 mmol/L, the insulin infusion rate should be reduced to 0.02–0.05 units/kg/h, coinciding with the addition of 5% dextrose infusion (1).

Whilst weak evidence has supported the traditional preference of low-dose over high-dose insulin, there is currently a lack of head to head studies guiding choice between fixed weight-based dosing over BGL-based sliding scale dosing.

#### Transition to Subcutaneous Insulin

On resolution of DKA and cessation of intravenous insulin infusion, rebound hyperglycaemia is a major issue (43, 66). Once an intravenous infusion is ceased its duration of action is only minutes, whilst the onset of basal subcutaneous insulin is 1–2 h and thus overlap of the two is an integral component of successful transition.

In 2007, a small study (*n* = 75) investigating the optimal insulin dose for transition from IV insulin infusion to subcutaneous insulin glargline, randomized patients to receive either 40%, 60%, or 80% of their total daily insulin requirement, calculated from the rate of insulin infused during the last 6 h of their insulin infusion, as insulin glargine (67). The study concluded that insulin glargine administered at 80% of the total daily insulin requirement resulted in the highest percentage of capillary blood glucose level within target glycaemic range of 4.4–8.3 mmol/L within the initial 24 h after transition (67).

A prospective randomised trial compared multidose insulin regimens demonstrated that split mixed insulin (NPH plus regular insulin) and basal bolus insulin regimen (glargine and glulisine) offered similar glycaemic control though basal bolus regimen was associated with a lower rate of hypoglycaemic events (15 vs. 41%) (1).

The ADA 2009 consensus statement recommend patients normally on subcutaneous insulin should be recommenced on their usual dose if this was typically adequate for glycaemic control, whilst insulin naive patients should be commenced on either a multidose insulin of 2–3 boluses daily or basal bolus regimen (1).

Current limited evidence suggest that transitioning from insulin infusion to subcutaneous insulin using 80% of the total insulin dose offers optimal glycaemic control, with nil evidence to guide recommendations for either a spit mixed or basal bolus regime.

### Glucose Infusion

Glucose infusion is recommended when blood glucose levels falls below 10–14 mmol/L (1, 5, 29, 68) to avoid hypoglycaemia (3, 5) and to reduce the development of ketosis (3, 5, 29). However, the choice of these thresholds is somewhat arbitrary.

Regarding the choice of fluid for maintenance of adequate glycaemic control, 5% dextrose was compared to 10% dextrose in a small RCT (*n* = 17). This study found that 10% dextrose resulted in significantly lower level of ketonaemia and a higher level of hyperglycaemia but did not result in any benefits in correction of acidosis or bicarbonate levels (36).

There is currently lack of evidence guiding threshold blood glucose levels for introduction of dextrose with no evidence suggesting a difference in outcomes between administration of dextrose 5 and 10%.

### Interpretation of DKA Correction Rate

Studies have traditionally used time to correction of DKA as a surrogate end point for patient outcomes, where resolution of DKA is defined as BGL < 11mmol/L and two of the following criteria: serum bicarbonate level ≥15 mmol/L, venous pH >7.3 and a calculated anion gap ≤12 mmol/L (2). Despite the assumption that faster correction of DKA leads to better outcomes with shorter intensive care and hospital admission, there have been no studies which specifically establish this relationship. A prospective trial (*n* = 114) concluded that very low-dose insulin administration and slow re-equilibration were the fundamental strategies in optimal management (69). The study used a basal insulin infusion of 1 unit/h, with a maximal decrease of blood glucose level of 3 mmol/L/h, reaching a mean blood glucose of 14 mmol/L at 12 h, whilst rehydrating with 1 L/h for the first 4 h. This approach resulted in no mortality or lasting deficiencies in a small cohort (69).

Studies addressing this issue have been performed in the paediatric population and one retrospective observational study (*n* = 67) found an insulin infusion rate of 0.05 units/kg/h resulted in more gradual reduction in effective plasma osmolality over the first 12 h, and a similar length of intensive care admission to patients who received insulin doses of 0.1 unit/kg/h (70). Similarly, a small open-label randomised trial (*n* = 50) also in the paediatric population found that standard insulin doses of 0.1 unit/kg/h compared to low dose 0.05 units/kg/h was associated with higher rates of hypoglycaemia (20 vs. 4%, *p* = 0.17), hypokalaemia (48 vs. 20%, *p* = 0.07), and cerebral oedema (4 vs. 0%), whilst time to reduction of blood glucose level and resolution of acidosis were similar (71).

Given these findings, we conclude that rapid resolution of DKA does not necessarily correlate with superior clinical outcomes. Guidelines do not address this issue, and further research is needed to explore this question.

### Use of Protocols

Limited evidence suggests that the use of a protocol, whether based on national guidelines or individual institution-formed guidelines leads to a better adherence to key treatment principles and improved patient outcome (4, 12, 46, 72–77).

Adherence rates of DKA management protocols were studied, in a retrospective review of 71 cases. The study found an average of 30% adherence rates to guidelines and the study concluded outcomes were similar amongst patients treated with and without guidelines (78) (Table 6).

**Table 6:** **Adherence rates to diabetic ketoacidosis management protocols**.

Study	Cases	End point	Performance rates (%)
Singh et al.—retrospectivereview (78)	*n* = 71	Timely initiation of intravenous fluid replacement	30
		Timely initiation of intravenous insulin	31
		Adequate intravenous fluids within the first 24 h	30

Devalia—retrospective case note review (4)	*n* = 46	DKA correctly diagnosed as per protocol	78
		Intravenous access and correct blood tests within the first hour of admission	100
		Appropriate fluid resuscitation	87
		Correct insulin prescribed	72
		Correct sliding scale	73
		Patients not requiring high dependency unit managed in the appropriate ward setting	89
		Patients requiring high dependency unit and appropriately referred	46

The uptake of a new DKA protocol was studied in a retrospective audit of 46 DKA episodes over a 4-month period in 2008, with adherence rates above 70% in the initial hour (Table 6), although this declined after 1 h following commencement of treatment, resulting in incorrect fluids being administered and neglect in monitoring electrolytes (4). The reasons for this deviation from protocol were unclear although it was postulated to be due to lack of defined parameters indicating need for HDU referral, use of physician clinical judgement and inefficient patient handover during patient ward transfer and handover between shifts (4, 78).

Other authors raised the view that strict adherence to protocols may not be the gold-standard, and that deviations from protocols may be beneficial or even necessary in some instances (72). Protocol use may be poor if they are perceived to lack credibility, have systemic bias, reduce physician autonomy, or if they are not seen to be applicable to all patients (4, 46, 72, 73).

Regarding protocol impact on DKA treatment outcomes, a retrospective study compared patients managed with and without local protocol, found that patients treated with a local protocol had a significantly shorter mean time to normalisation of serum bicarbonate, lower incidences of hypokalaemia, and lower incidence of hypoglycaemia, and no significant difference in total insulin dose (11) (Table 7).

**Table 7:** **Impact on outcomes when treating diabetic ketoacidosis according to protocol**.

Study	Arms	End points	Findings	*p*-Value
			Protocol vs. non-protocol arms respectively	
Thuzar et al.—retrospective study (11)	Protocol (*n* = 35) No protocol (*n* = 36)	Mean length of hospitalisation	37.9 vs. 49.2 h	0.01
		Mean time to normalisation of serum bicarbonate	15.1 vs. 24.6 h	0.01
		Incidences of hypokalaemia	28.6 vs. 52.8%	0.038
		Incidence of hypoglycaemia	8.6 vs. 28%	0.036

Hara et al.—retrospective review (12)	Protocol (*n* = 113) Non-protocol (*n* = 143)	Mean difference in time to resolution	−9.2 h (95% CI: 4.7–13.7)	<0.01
		Frequency of hypoglycaemia	8 vs. 11.2%	0.259
		Frequency of hypokalaemia	30.1 vs. 32.3%	0.413
		Mean difference in length of hospital stay	−2.04 days (95% CI: 0.61–3.46)	0.005

In 2011, a retrospective review of patients treated for DKA or HHS found that patients treated according to protocol had a shorter mean duration of time to resolution, and no difference in frequency of hypoglycaemia or hypokalaemia (12) (Table 7).

The limited current evidence suggest that adherence to protocols is poor to modest. Despite limited adherence to strict protocol fidelity, there appear to be improvements in patient outcomes (4, 12, 46, 72–74). Protocols are likely to be beneficial as a guide to nursing and medical staff with the caveat that deviation from protocol to tailor to individual circumstances may be warranted.

## Limitations

We note this is a narrative review, not a systematic review, hence potential remains for further synthesis of identified evidence. Although we considered several key aspects of DKA management, there are many other aspects of treatment that were not formally reviewed. These aspects include the roles and implications of separate emergency department, ward, and intensive care unit protocols; severity assessment; special patient groups with comorbidities including drug and alcohol use; age-based fluid replacement, patient education and other preventative strategies, and complications of DKA. Euglycaemic DKA was also not reviewed.

## Discussion and Conclusion

On review, the strength and breadth of original evidence is limited. Current weak evidence suggests crystalloids are preferred over colloids. There is no clear evidence for superiority of a given crystalloid nor for a given rate of rehydration.

Low-dose insulin has limited evidence for superior safety over high-dose insulin. Administering bolus insulin prior to low-dose insulin infusions <0.14 units/kg/h may obviate the need for additional insulin supplementation. Intravenous regular insulin infusions are traditionally preferred, with emerging evidence for non-inferiority of subcutaneous insulin and with no head to head comparisons between fixed weight-based versus blood glucose-based insulin dosing regimens. Clear evidence is lacking for timing of initiation and titration of dextrose infusions, in addition to replacement of potassium, phosphate, bicarbonate. Traditionally, studies have used mean time to resolution of DKA as favourable end points, although this correlation has no supporting evidence.

Few studies consider patient outcome using various protocols, with improvement in outcomes.

Currently, there is a major deficiency of strong evidence for the optimal management of DKA in the adult population with a mixture of consensus opinion and weak evidence providing the framework within which physicians manage DKA. There are inherent difficulties in conducting robust studies and these include low rates of patient admissions with DKA, large numbers of patients required to show differences and the multiple confounding variables contributing to outcomes. Despite this, all components of DKA management would benefit from prospective RCTs and there is vast potential for further studies to elucidate or establish a more robust evidence-based practice for best patient outcomes.

## Author Contributions

TT and AP—selection of articles and drafting of manuscript. TT, AP, EE, AW, JZ, JM, and RB—all authors contributed substantially to the conception and design of the work, reviewing, final approvals of the version submitted, and agree to be accountable for accuracy and integrity of content.

## Conflict of Interest Statement

The authors declare that the research was conducted in the absence of any commercial or financial relationships that could be construed as a potential conflict of interest.
