# Corrigendum: Review of Evidence for Adult Diabetic Ketoacidosis Management Protocols

**DOI:** 10.3389/fendo.2017.00185

**Published:** 2017-07-31

**Authors:** Tara T. T. Tran, Anthony Pease, Anna J. Wood, Jeffrey D. Zajac, Johan Mårtensson, Rinaldo Bellomo, Elif I. Ekinci

**Affiliations:** ^1^Department of Endocrinology, Austin Health, Melbourne, VIC, Australia; ^2^Department of Medicine, Austin Health, University of Melbourne, Melbourne, VIC, Australia; ^3^Department of Intensive Care, Austin Health, Melbourne, VIC, Australia; ^4^Menzies School of Health Research, Darwin, NT, Australia

**Keywords:** diabetic ketoacidosis, diabetes, insulin, rehydration, hypoglycemia, hypokalemia, metabolic acidosis, protocol

An author name was incorrectly spelled as Elif I. I Ekinci. The correct spelling is Elif I. Ekinci. The authors apologize for this error and state that this does not change the scientific conclusions of the article in any way.

The original article has been updated.

## Conflict of Interest Statement

The authors declare that the research was conducted in the absence of any commercial or financial relationships that could be construed as a potential conflict of interest.

